# Advancements in Retinitis Pigmentosa: The Path Toward Personalized Treatment and Vision Restoration

**DOI:** 10.2174/0113892029391676250825112617

**Published:** 2025-08-29

**Authors:** Komal, Lovekesh Singh, Subrahmanya Sarma Ganti

**Affiliations:** 1 Department of Pharmaceutics, ISF College of Pharmacy, Moga, Punjab 142001, India;; 2 Department of Pharmacology, ISF College of Pharmacy, Moga, Punjab 142001, India;; 3 Department of Pharmaceutical Analysis, ISF College of Pharmacy, Moga, Punjab 142001, India

## 1 INTRODUCTION

Retinitis Pigmentosa (RP) is a diverse, genetically and phenotypically heterogeneous retinal degenerative disorder, which affects 1 out of every 4,000 people worldwide. RP is still an incurable disease, even though genetic and regenerative medicine show several promising developments. Earlier studies have tended to provide either limited reviews of specific therapeutic developments or general descriptive surveys that do not involve any critical appraisal [1]. An integrated and multimodal approach to RP management can be proposed as a system-based approach to individual treatment. We are striving to integrate the knowledge gained in molecular genetics, imaging tools, non-genetic treatments, and individualized diagnostics into a coordinated approach that facilitates clinical translation and patient stratification [2]. Fig. (**1**) shows a comparison of a healthy retina and a retina affected by retinitis pigmentosa (RP).

## 2 PATHOPHYSIOLOGY OF RETINITIS PIGMENTOSA

The gradual death of rod cells, which in turn initiates the degeneration and eventual death of cone cells, has traditionally been considered the primary manifestation of RP. However, new studies have focused on several additional contributing factors:

- Microglial activation and chronic inflammation
- Oxidative stress and mitochondrial abnormalities
- Dysregulation of Müller glia homeostasis
- Irregular choroidal-retinal vasculature

These changes can now be imaged and measured using advanced imaging technologies, such as Optical Coherence Tomography Angiography (OCT-A), Adaptive Optics Scanning Light Ophthalmoscopy (AOSLO), and multifocal Electroretinography (mfERG). These tools contribute to individualized functional phenotyping [3, 4].

## 3 ACCURATE IMAGING AND PREDICTION IN BIOMARKERS

The progression of the disease and response to treatment can be monitored in real time, non-invasively, using imaging technologies. It is important to incorporate these tools into clinical pathways:

- OCT-A allows monitoring of choroidal and retinal perfusion losses.
- AOSLO enables mapping of cone density at cellular resolution.
- Systemic vascular contributions are assessed through Ophthalmic Artery Doppler examination.
- Profiling of Flammer syndrome assists in detecting autonomic dysfunction in certain patients.

These modalities are underlinked with a strong potential for patient stratification, especially when integrated with genetic data.

## 4 GENE THERAPY: CORRECTION TO CUSTOMIZATION

The newest gene therapies, including base editing, prime editing, next-generation capsids, and exosome-based delivery, provide even more precise and tissue-specific techniques. Although these methods are expected to be more effective and demonstrate reduced immunogenicity, issues such as effective delivery, off-target effects, immunogenicity, and large-scale production still hinder progress. Table **1** shows the emerging gene therapy techniques for retinitis pigmentosa, mechanisms, strengths, and challenges.

Efforts are now focused on designing immune-evasive, photoreceptor-targeting vectors with minimal toxicity and scalable manufacturing. It is a milestone in ocular gene therapy that the drug Luxturna (voretigene neparvovec) against RPE65-mediated RP has been approved [9, 10]. Nonetheless, the majority of RP variants do not have approved therapies because each is genetically heterogeneous, delivery is hindered, and immune complications are involved [11, 12].

## 5 GENE THERAPY: COMPLEMENTARY TREATMENT APPROACHES

Due to late-stage RP, gene correction is not sufficient; therefore, non-genetic interventions are crucial [13]. Optogenetics aims to re-sensitize remaining retinal ganglion or bipolar cells by introducing light-sensitive proteins, whereas retinal implants, such as PRIMA and Argus II, bypass damaged photoreceptors to restore some vision [14]. Nutraceuticals (such as DHA, NAC, and lutein) have neuroprotective effects, as well as broadly protective anti-inflammatory properties [15]. New metabolic therapies (such as ketogenic diets and profiling of the gut microbiome) are used to combat retinal inflammation, and physical therapy (such as posture correction, post-concussion rehabilitation, and Doppler-based vascular flow exercises) is utilized to improve ocular perfusion and overall retinal well-being [16].

Fig. (**2**) illustrates the emerging therapies for Retinitis Pigmentosa, including gene replacement and stem cell therapy administered *via * subretinal and intravitreal injections.

## 6 A SUGGESTED MODEL OF CUSTOMIZED RETINITIS PIGMENTOSA ADMINISTRATION

We suggest a three-level structure that could be used to individualize RP treatment by combining the profiling of molecular genetics, retinal phenotype, and systemic-functional profile.

The first level involves classifying genetic variants (dominant, recessive, X-linked, digenic) and the pathogenetic mechanisms of the disease (such as ciliary dysfunction or phototransduction errors).

The second level evaluates retinal characteristics, including cone density *via * AOSLO, ellipsoid zone integrity *via * OCT, and microvascular density using OCT-A.

The third level assesses global and functional parameters, including vascular status *via * Doppler flow, inflammation, and metabolic risk markers (*e.g*., CRP, gut microbiome, and Flammer syndrome), as well as functional vision measurements, such as contrast sensitivity and mobility performance.

Such a combined approach allows patients to be stratified into therapeutic groups, including gene therapy, prosthesis recipient, neuroprotective emphasis, or a multimodal approach, based on the accompanying flowchart and scoring scheme.

## 7 TRANSLATION AND READY CLINICAL TRIAL METRICS

Lack of clarity regarding endpoints has been the albatross of RP trials; hence, pre-determined efficacy outcomes, including cone survival (OCT), fixation stability (microperimetry), and real-world mobility tests, have been advised. The recruitment process should follow the proposed stratification structure and be reported accordingly, adhering to the STROBE, PRISMA, and Cochrane Risk of Bias 2.0 guidelines, as well as PROSPERO registration for meta-reviews, to enhance transparency and reliability.

## 8 CHALLENGES AND FUTURE DIRECTIONS

The problems associated with RP treatment include the lack of scalable, affordable, and regulated delivery modalities. There are complications related to immune responses to tools, such as CRISPR/Cas9 and AAV capsids. New treatment options include mRNA-based therapies, programmable epigenetics, and AI-assisted imaging. A crucial aspect of clinical translation is the development of a phased roadmap, spanning from preclinical projects to equitable access [17].

## CONCLUSION

Retinitis Pigmentosa remains a complex and challenging disease, yet the path to effective treatment is becoming clearer than ever. Through a stratified, patient-centered combination of gene editing, prosthetic augmentation, personalized imaging, and systemic modulation, clinicians and researchers can significantly improve the course of care. Rather than viewing innovation as an isolated phenomenon, the future of RP therapy lies in multimodal, orchestrated, and data-driven personalization, guided by robust translational ethics.

## AUTHORS’ INSIGHTS

- The management of Retinitis Pigmentosa requires a combination of treatments rather than isolated approaches.
- Gene therapy is not bulletproof; systemic, neural, and prosthetic solutions have to be combined with it.
- The importance of predictive imaging and vascular diagnostics is not yet fully realized and should become a key factor in the clinical decision-making process.
- Better treatment stratification will be achieved through personalized algorithms that incorporate genetic, structural, and functional data.
- Realizing real-world results requires cross-disciplinary collaboration, through which genetics, ophthalmology, neurology, bioengineering, and rehabilitation collaborate.

## Figures and Tables

**Fig. (1):**
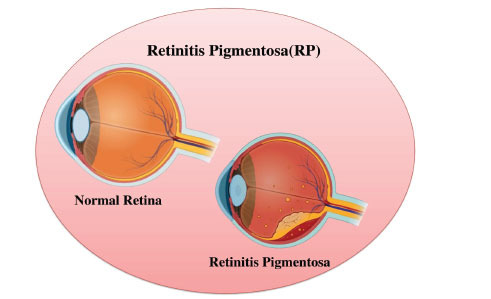
Comparison of a healthy retina and a retina affected by retinitis pigmentosa (RP).

**Fig. (2):**
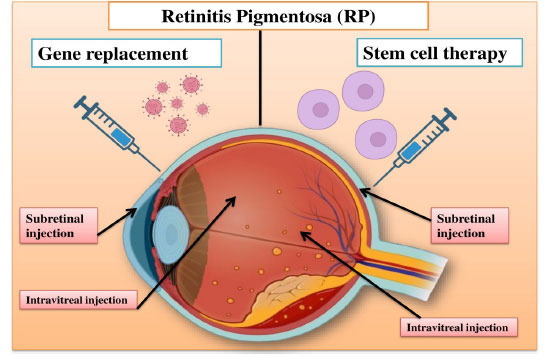
Emerging therapies for retinitis pigmentosa: Gene replacement and stem cell therapy *via * subretinal and intravitreal injections.

**Table 1:** Emerging gene therapy techniques for retinitis pigmentosa: Mechanisms, strengths, and challenges.

**Technique**	**Mechanism**	**Strengths**	**Challenges**	**References**
Base Editing	Direct conversion of DNA bases	High precision	Delivery & off-target risk	[5]
Prime Editing	RNA-guided reverse transcription	Versatile edit types	Limited *in vivo* validation	[6]
Next-Gen Capsids	Modified AAV/lipid NPs	Tissue-specific delivery	Immune response, durability	[7]
Exosomes	Natural vesicles for gene delivery	Low immunogenicity	Production scalability	[8]

## LIST OF ABBREVIATIONS

AOSLO: Adaptive Optics Scanning Light Ophthalmoscopy
mfERG: multifocal Electroretinography
OCT-A: Optical Coherence Tomography Angiography
RP: Retinitis Pigmentosa
